# Grey Matter Atrophy in Multiple Sclerosis: Clinical Interpretation Depends on Choice of Analysis Method

**DOI:** 10.1371/journal.pone.0143942

**Published:** 2016-01-08

**Authors:** Veronica Popescu, Menno M. Schoonheim, Adriaan Versteeg, Nimisha Chaturvedi, Marianne Jonker, Renee Xavier de Menezes, Francisca Gallindo Garre, Bernard M. J. Uitdehaag, Frederik Barkhof, Hugo Vrenken

**Affiliations:** 1 Department of Radiology and Nuclear Medicine, Neuroscience Campus Amsterdam (NCA), VU University Medical Center, Amsterdam, The Netherlands; 2 Department of Anatomy and Neurosciences, Neuroscience Campus Amsterdam (NCA), VU University Medical Center, Amsterdam, The Netherlands; 3 Department of Epidemiology and Biostatistics, VU University Medical Center, Amsterdam, The Netherlands; 4 Department of Neurology, Neuroscience Campus Amsterdam (NCA), VU University Medical Center, Amsterdam, The Netherlands; 5 Department of Physics and Medical Technology, Neuroscience Campus Amsterdam (NCA), VU University Medical Center, Amsterdam, The Netherlands; University Hospital Basel, SWITZERLAND

## Abstract

**Background:**

Studies disagree on the location of grey matter (GM) atrophy in the multiple sclerosis (MS) brain.

**Aim:**

To examine the consistency between FSL, FreeSurfer, SPM for GM atrophy measurement (for volumes, patient/control discrimination, and correlations with cognition).

**Materials and Methods:**

127 MS patients and 50 controls were included and cortical and deep grey matter (DGM) volumetrics were performed. Consistency of volumes was assessed with Intraclass Correlation Coefficient/ICC. Consistency of patients/controls discrimination was assessed with Cohen’s d, t-tests, MANOVA and a penalized double-loop logistic classifier. Consistency of association with cognition was assessed with Pearson correlation coefficient and ANOVA. Voxel-based morphometry (SPM-VBM and FSL-VBM) and vertex-wise FreeSurfer were used for group-level comparisons.

**Results:**

The highest volumetry ICC were between SPM and FreeSurfer for cortical regions, and the lowest between SPM and FreeSurfer for DGM. The caudate nucleus and temporal lobes had high consistency between all software, while amygdala had lowest volumetric consistency. Consistency of patients/controls discrimination was largest in the DGM for all software, especially for thalamus and pallidum. The penalized double-loop logistic classifier most often selected the thalamus, pallidum and amygdala for all software. FSL yielded the largest number of significant correlations. DGM yielded stronger correlations with cognition than cortical volumes. Bilateral putamen and left insula volumes correlated with cognition using all methods.

**Conclusion:**

GM volumes from FreeSurfer, FSL and SPM are different, especially for cortical regions. While group-level separation between MS and controls is comparable, correlations between regional GM volumes and clinical/cognitive variables in MS should be cautiously interpreted.

## Introduction

Brain atrophy in multiple sclerosis (MS) can be quantified and monitored over time with reliable and reproducible techniques [1] Especially grey matter (GM) atrophy seems to be correlated to clinical and neuropsychological measures [2], and it also becomes more prominent in advanced disease stages [3,4]. GM atrophy seems to be unevenly distributed across the brain in MS, with possible preference for insular cortex and thalamus, but studies disagree regarding not only the anatomical distribution of GM atrophy [5–8], but also regarding the clinical correlations of GM atrophy [9–13]. Drawing a conclusion from the body of published studies is difficult due to the heterogeneity of patient groups, and of the software packages used for segmentation. A study comparing methods directly in MS is so far lacking.

The goal of this study was to examine the consistency of GM atrophy measurements using three software (FSL, FreeSurfer and SPM) used frequently in MS GM atrophy literature, in a large cohort of MS patients with similar disease duration. We analyzed for each structure separately: the consistency between methods for regional GM volumes, consistency of the patients/controls discrimination, and consistency of associations with neuropsychological measures.

## Materials and Methods

### Descriptives and clinical measures

As part of their 6-years follow-up for an ongoing inception cohort study (PRESTO) in which MS patients were included six years prior, just before or at diagnosis, subjects underwent an MRI scan between October 2010 and December 2012 [14]. Age-, gender- and education-matched healthy controls from the Amsterdam area were also recruited from existing databases. The research was approved by the authors' Institutional Review Board (Medisch Ethische Toetsingscommissie of the VU University Medical Center, Amsterdam, The Netherlands) and all clinical investigation was conducted according to the principles expressed in the Declaration of Helsinki. Written informed consent was obtained from the participants, as indicated in the Methods section of the manuscript. Physical disability was measured using the Expanded Disability Status Scale (EDSS) and was relatively mild (median EDSS 2.0, range 0.0–6.0). Patients were relapse-free and without steroid treatment for at least 2 months.

Subjects underwent a comprehensive neuropsychological assessment of seven cognitive domains (executive functioning, verbal memory, information processing speed, visuospatial memory, working memory, attention, and psychomotor speed) [14] and an “average cognition” Z score was calculated by averaging Z scores of all separate domains, as previously described. This average cognition Z-score will be referred to as "cognition".

### Magnetic resonance imaging

Subjects received structural 3T-MR scans on the same MRI scanner (GE Signa HDXT, V15M), using the same scanning protocol including: sagittal 3D-T1 FSPGR sequence (TR = 7.82ms, TE = 3ms, TI = 450ms, FA = 12, voxel size = 0.98x0.98x1mm^3^), sagittal 3D-FLAIR (TR = 8000ms, TE = 128ms, TI = 2348ms, FA = 90, voxel size = 0.98x0.98x1.2mm^3^) and axial 2D dual-echo PD-T2 (TR = 9680ms, TE = 22/112ms, FA = 90, voxel size = 0.6x0.6x3mm). All scans were visually inspected for quality.

### Image preprocessing

The analyses were performed using the cluster computer of the Neuroscience Campus Amsterdam running under Linux CentOS (http://www.neurosciencecampus-amsterdam.nl/en/news-agenda/news-archive/2013/NCAGRID-cluster-computer.asp). Brain volumes were analyzed on the 3D-T1 sequence, after removal of excessive neck slices and lesion-filling.

**Fig 1:**
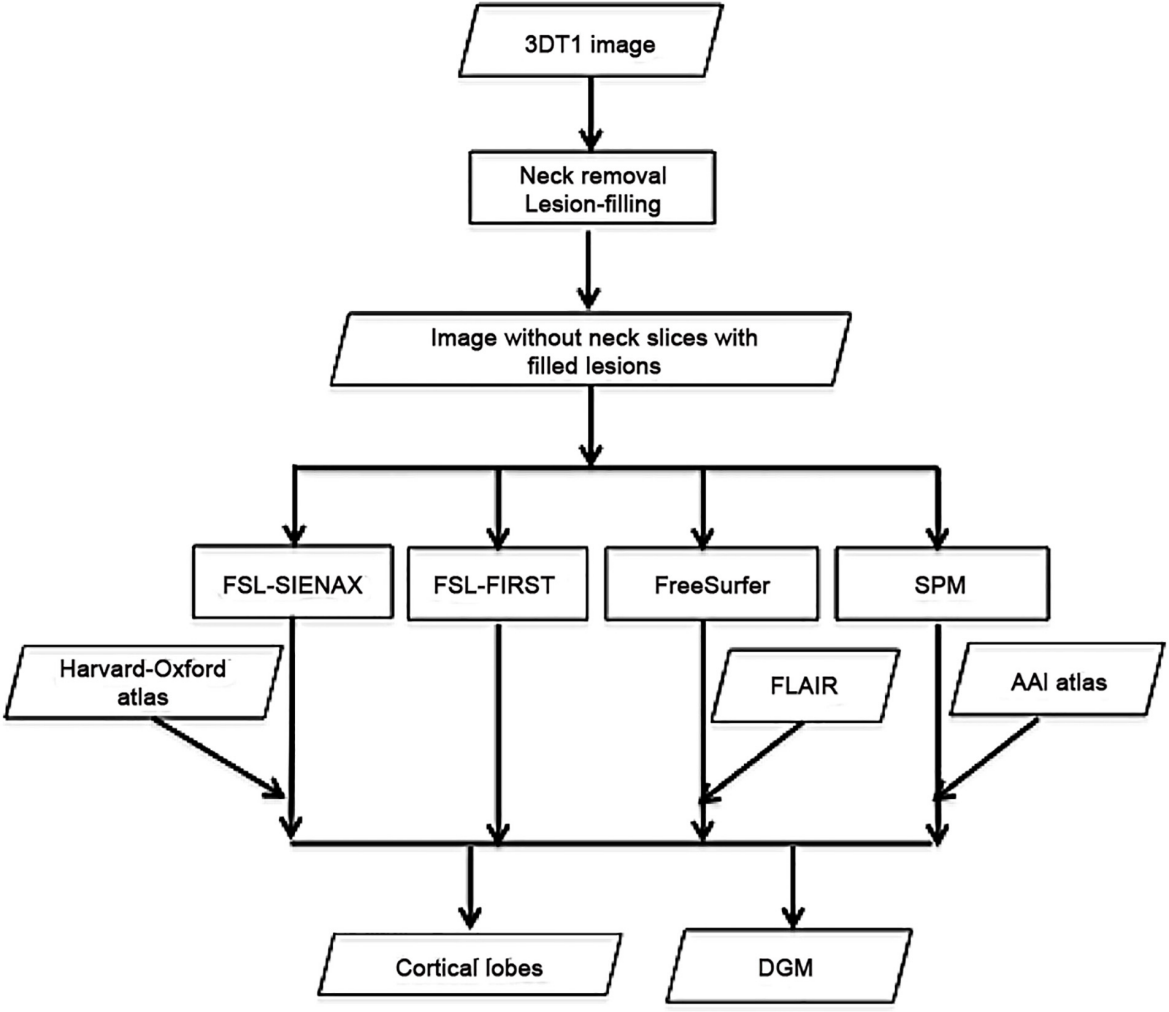
Workflow.

The lesions were first automatically segmented on the 3D-FLAIR images using an in-house developed kNN-TTP technique [15], the resulting lesion masks being registered to the 3DT1 images. These lesion masks were used for lesion-filling with LEAP [16], in order to diminish the effect of the white matter (WM) lesions on the segmentation of the GM. To avoid filling the DGM with WM intensities, the DGM lesions were removed from the masks before lesion-filling. The workflow is depicted in Fig 1.

The T1-hypointense and T2-hyperintense lesions were marked on the T1-images and the dual-echo images respectively by an experienced rater, and lesion volumes were measured using a local thresholding technique using Alice software (Hayden Image Processing Group/Parexel International Corp, Waltham, MA).

### Image processing: GM volumetrics

The following regional GM segmentation algorithms were used for lobar GM volumes and DGM volumes (details of the implementation are given in the sections below).

FSL: SIENAX [17] registers the individual scan to the standard space brain (derived from the MNI-152 standard image), using the skull as a scaling constraint, and uses FAST for automated tissue-type segmentation. The Harvard-Oxford atlas delivered with FSL was non-linearly registered (using FNIRT) and used on the GM partial volume estimates for calculating the lobar cortical GM volumes. Volumetry of DGM structures was performed using FIRST [18], which models the outer surface of each DGM structure as a mesh, and finally, it assigns each voxel in the image the appropriate label to indicate of which structure it is a part, taking into account local variations in structure surface shape, as well as the presence of neighboring structures.

FreeSurfer [19,20] performs cortical (surface-based) analysis for the cortical thicknesses and volumes, and volume analysis for the DGM structures in the native space. FreeSurfer also outputs the results within the cerebral lobes (left and right): frontal, temporal, insula, cingulate, parietal, occipital. FreeSurfer was used both with and without the FLAIR images for pial-surface refinement (https://surfer.nmr.mgh.harvard.edu/fswiki/ReleaseNotes).

SPM8 (Statistical Parametric Mapping Functional Imaging Laboratory, University College London, London, UK) accessed through the VBM8 toolbox (http://dbm.neuro.uni-jena.de/vbm/), automatically identifies and quantifies per voxel GM, WM, and CSF in all scans. The AAL atlas delivered as part of SPM toolboxes was registered with FSL-FLIRT and FSL-FNIRT to the native scans and then used for calculating the lobar GM volumes. This atlas does not map the nucleus accumbens.

### Inspection of segmentation quality

All outputs were inspected for quality, and excluded if gross errors were visible.

### Influence of atlas

In order to separate the influence of the segmentation algorithm from that of the atlases used in this study for each algorithm, the volumes of the cortical lobes were also calculated from the FSL and SPM segmentation by overlaying for each subject the FreeSurfer brain lobes parcellation on the GM partial volume estimates. Here, to avoid exclusion of GM voxels due to partial volume effects in the FSL and SPM segmentations, the lobar parcellation for each subject was dilated using a 3x3x3 kernel four times, using modal dilation to avoid intersections between structures. As an extra analysis, the AAL atlas registered to the SPM segmentation was also dilated using a 3x3x3 kernel four times, using modal dilation to avoid intersections between structures.

### Image processing: Voxelwise and vertexwise statistical analyses

Voxel-based morphometry (SPM-VBM8 toolbox and FSL-VBM) and vertex-wise FreeSurfer were used to map, and compare between these three methods, the local GM differences between MS patients and controls. In FSL-VBM [21], the GM images were registered to the MNI-152 standard space and averaged to create a left-right symmetric, study-specific grey matter template. In SPM-VBM8 toolbox, a DARTEL template of the GM [22] of all scans was created by nonlinearly aligning the GM images to a common space of 1.5x1.5x1.5mm^3^. For both methods an 8-mm Gaussian kernel was used for smoothing, as frequently applied in literature. Voxelwise GLM was applied using parametric testing for SPM and permutation-based non-parametric testing for FSL, correcting for multiple comparisons across space.

Vertex-wise FreeSurfer analysis was used to create a study-specific template, and vertex-wise comparisons were performed after smoothing using a 10-mm FWHM Gaussian kernel, as frequently applied in literature.

As gender was previously shown to greatly influence atrophy in this cohort of MS patients [14], the voxel-wise and vertex-wise statistical comparisons between patients and controls aimed at localizing GM differences were run both with and without gender and age as covariates.

### Statistical analysis

The consistency of GM volumes was assessed in SPSS20 with the Intraclass Correlation Coefficient (ICC) (consistency) for each measured lobar GM and DGM volume.

For the consistency of patient/control discrimination we assessed both each anatomical structure separately and also the consistency with which all structures contributed to patient-control discrimination in a combined model. The consistency of patient/control discrimination for each anatomical structure separately was assessed with Cohen’s d, t-tests, and MANOVA with age and gender included in SPSS20. The consistency with which all structures contributed to patient-control discrimination in a combined model was assessed with a penalized double-loop logistic classifier [23,24] in R for each segmentation software separately. The double-loop classifier used L1 penalization based logistic regression for classifying the binomial response. The model parameters were estimated using the inner loop cross-validation and the prediction accuracy for the estimated model parameters was estimated using the outer cross-validation loop. This division of the classifier into two cross-validation loops was done to avoid over-fitting and to reduce the number of false positives.

The consistency of the association with cognition was assessed in SPSS20 with Pearson correlation coefficient between normalized lobar GM and DGM structure volumes and cognition. Additionally, multiple regression was also performed with age and gender included, using cognition as outcome measure.

Significance was considered at p < .05, after appropriate correction for multiple comparison.

Statistical analyses for FSL-VBM were performed in FSL using Randomise, for SPM-VBM in SPM, and FreeSurfer vertex-wise statistics were performed in FreeSurfer both with and without age and gender as covariates.

## Results

A total of 127 RRMS patients (91 women) and 50 healthy controls (30 women) were included. Clinical data are detailed in Table 1. In order to use the same images for all analyses, 33 scans of patients (but no controls) had to be excluded after visual inspection for segmentation errors. The errors were as follows: for 17 patients the cortex was severely underestimated in FreeSurfer (because the pial surface ran through the cortex), for 15 patients gyri were missed at the segmentation in FreeSurfer, and for one patient with severe pathology and asymmetric ventricles the DGM segmentation failed for both FreeSurfer and FSL-FIRST (only the structures on the right side were correctly segmented). After excluding these subjects, 94 patients and 50 controls remained in the analyses.

**Table 1:** Clinical and radiological characteristics of all patients’ groups in this study.

	Controls	Patients Included	Patients with Image Errors	p-value (patients in the study and patients excluded)
No of patients	50	94	33	
Women	36	72	19	.038
Mean age (y) (standard deviation)	43 (9)	39 (8)	42 (9)	.044
Mean disease duration (y) (standard deviation)	-	7 (2)	8 (3)	.103
Right-handed		80	25	.056
EDSS*		2 (1)	2.5 (1)	.597
NBV*	1.53 (0.06)	1.49 (0.06)	1.47 (0.07)	.143
NGMV*	0.83 (0.05)	0.82 (0.04)	0.8 (0.05)	.027
Median T2LV (mL) (IQR)*	-	3 (3.3)	5.5 (5.9)	.031

The columns represent the results for the: patients and the healthy controls. DD = disease duration. DMT = disease modifying treatment. NBV = normalized brain volume. NGMV = normalized grey matter volume. T2LV = T2 lesion volume.

* Data reported as (median (IQR))

The differences between the excluded patients and the patients still in the study are presented in Table 1. The excluded patients group contained more men; the mean age was higher, with lower total grey matter volume and higher WM lesion volumes.

Using t-tests MS patients scored significantly lower than controls on most cognitive domains: executive functions (p = .001), information processing speed (p = .002), working memory (p < .001), attention (p < .001), psychomotor speed (p = .001) and general cognition (p < .001).

**Fig 2:**
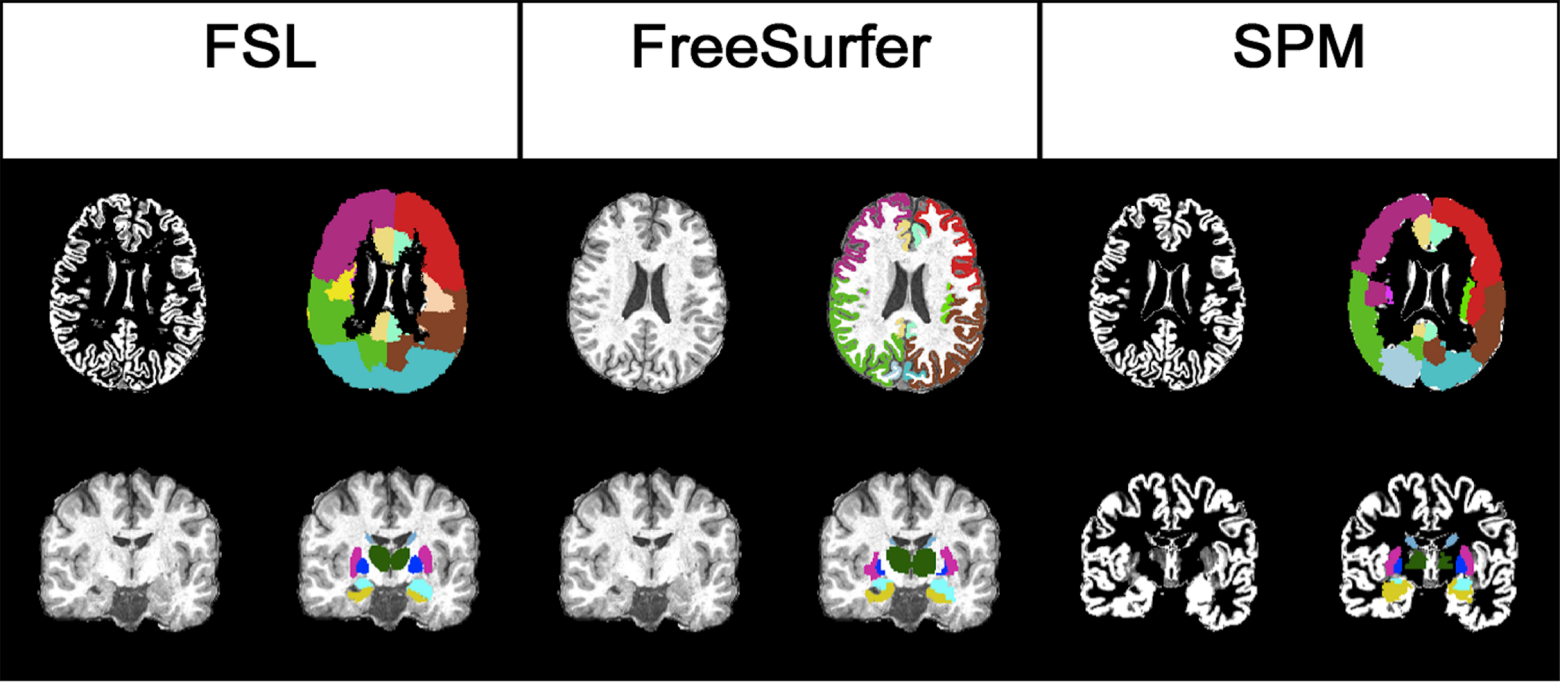
Segmentation obtained through different software packages with the lobes (upper row) and DGM (lower row) overlay, from left to right: FSL (FSL GM segmentation and GM segmentation+overlay), FreeSurfer (brain and brain+overlay) and SPM (SPM GM segmentation and GM segmentation+overlay).

A segmentation example for all techniques is presented in Fig 2.

The normalized brain volumes from FSL-SIENAX were lower in patients compared to controls (1.49±0.06L vs. 1.53±0.07L, p < .001), as well as the normalized grey matter volumes from FSL (0.81±0.05L vs. 0.83±0.05L, p = .02). Tables 2 and 3 present the regional GM volumes obtained with each method. Only DGM volumes tended to be smaller in MS compared to controls for all methods. As expected, adding FLAIR pial surface refinement did not alter FreeSurfer results for DGM volumes, while it did change FreeSurfer cortical regional GM volumes.

**Table 2:** Lobar GM volumes (mL) with all software (mean(SD)).

	FSL		FreeSurfer FLAIR		FreeSurfer no FLAIR		SPM	
	Patients	Controls	Patients	Controls	Patients	Controls	Patients	Controls
L-Insula	6.5 (0.7)	6.5 (0.7)	6.6 (0.7)	6.5 (0.7)	6.7 (0.8)	6.5 (0.7)	7.5 (0.9)	7.5 (0.9)
R-Insula	6 (1)	6 (0.6)	6.7 (0.9)	6.7 (0.8)	6.7 (0.9)	6.7 (0.8)	7.3 (0.9)	7.3 (0.8)
L-Parietal	39 (6)	39 (4)	56 (6)	55 (6)	57 (6)	56 (6)	44 (5)	43 (5)
R-Parietal	43 (7)	42 (5)	57 (6)	56 (6)	58 (6)	57 (6)	42 (5)	41 (5)
L-Temporal	59 (6)	59 (6)	53 (6)	53 (6)	56 (6)	55 (6)	47 (6)	46 (5)
R-Temporal	60 (7.5)	59 (6)	52 (6)	51 (5)	54 (6)	54 (6)	52 (6)	51 (6)
L-Occipital	50 (7)	50 (6)	23 (3)	22 (3)	23 (3)	23 (3)	45 (5)	44 (5)
R-Occipital	55 (8)	55 (6)	23 (3)	22 (3)	23 (3)	23 (3)	40 (4.7)	40 (5)
L-Cingulate	8 (1)	7.8 (0.9)	10 (1.4)	9.7 (1.5)	10 (1.4)	10 (1.5)	12 (1.6)	12 (1.5)
R-Cingulate	11 (1.6)	11 (1.4)	9.5 (1)	9.5 (1.5)	9.7 (1)	9.8 (1.5)	12 (1.6)	12 (1.6)
L-Frontal	86 (12)	85 (10)	86 (9)	83 (9.6)	87 (9)	85 (10)	77 (9)	75 (9.4)
R-Frontal	91 (13)	89 (11)	85 (9)	83 (9.6)	87 (10)	84 (9.8)	78 (9)	76 (9)

**Table 3:** DGM volumes (mL) with all software (mean(SD)).

	FSL		FreeSurfer		SPM	
	Patients	Controls	Patients	Controls	Patients	Controls
L—Accumbens	0.5 (0.1)	0.6 (0.1)	0.5 (0.1)	0.6 (0.1)	-	-
R—Accumbens	0.4 (0.1)	0.4 (0.1)	0.6 (0.1)	0.6 (0.1)	-	-
L—Amygdala	1.3 (0.2)	1.3 (0.2)	1.4 (0.2)	1.5 (0.2)	1 (0.1)	1 (0.1)
R—Amygdala	1.2 (0.2)	1.3 (0.2)	1.5 (0.2)	1.6 (0.2)	1 (0.1)	1 (0.1)
L—Caudate	3.3 (0.5)	3.5 (0.4)	3.4 (0.5)	3.6 (0.4)	3.4 (0.6)	3.7 (0.4)
R—Caudate	3.5 (0.4)	3.7 (0.5)	3.6 (0.5)	3.8 (0.5)	3.6 (0.6)	3.9 (0.5)
L—Hippocampus	3.6 (0.5)	3.8 (0.5)	4 (0.5)	4.2 (0.4)	3.8 (0.4)	3.9 (0.4)
R—Hippocampus	3.8 (0.5)	3.9 (0.4)	4 (0.5)	4.3 (0.5)	3.8 (0.4)	3.9 (0.3)
L—Pallidum	1.6 (0.2)	1.7 (0.1)	1.1 (0.2)	1.3 (0.2)	0.3 (0.06)	0.2 (0.06)
R—Pallidum	1.6 (0.2)	1.8 (0.2)	1.3 (0.2)	1.5 (0.2)	0.2 (0.05)	0.2 (0.06)
L—Putamen	4.7 (0.7)	4.9 (0.6)	5 (0.7)	5.2 (0.8)	3 (0.6)	3.3 (0.5)
R—Putamen	4.7 (0.6)	4.8 (0.5)	4.9 (0.7)	5 (0.6)	2.7 (0.6)	3 (0.5)
L—Thalamus	7.5 (0.8)	8 (0.8)	7.4 (1)	7.6 (0.9)	2.3 (0.5)	2.7 (0.4)
R—Thalamus	7.3 (0.8)	7.7 (0.8)	6.3 (0.8)	6.6 (0.8)	2.5 (0.5)	2.9 (0.5)

### Consistency of volumes

Table 4 provides the ICC values between methods for each GM regional volume. For the cortex, the agreement was higher, with most lobes having high agreement (ICC>.7) for all pairs of methods. FSL showed lower agreement with both FreeSurfer and SPM for the occipital lobes and the right insula. The agreement between FreeSurfer (both with and without FLAIR) and SPM was high for all cortical regions, with all ICC>0.7 except the left occipital lobe.

**Table 4:** Intraclass Correlation Coefficient values.

	FSL-FreeSurfer FLAIR	FSL-FreeSurfer no FLAIR	FSL-SPM	FreeSurfer FLAIR- SPM	FreeSurfer no FLAIR- SPM
L-Insula	0.923	0.915	0.915	0.896	0.892
R-Insula	0.463	0.46	0.529	0.838	0.835
L-Parietal	0.771	0.779	0.8	0.88	0.894
R-Parietal	0.75	0.763	0.778	0.83	0.86
L-Temporal	0.951	0.963	0.957	0.942	0.951
R-Temporal	0.86	0.875	0.865	0.917	0.952
L-Occipital	0.512	0.524	0.786	0.689	0.701
R-Occipital	0.464	0.468	0.688	0.767	0.782
L-Cingulate	0.718	0.717	0.775	0.796	0.799
R-Cingulate	0.772	0.776	0.866	0.803	0.808
L-Frontal	0.84	0.846	0.843	0.952	0.96
R-Frontal	0.841	0.843	0.846	0.946	0.959
L—Accumbens	0.654	0.654			
R—Accumbens	0.444	0.444			
L—Amygdala	0.386	0.386	0.375	0.64	0.64
R—Amygdala	0.197	0.197	0.318	0.519	0.519
L—Caudate	0.908	0.908	0.896	0.879	0.879
R—Caudate	0.938	0.938	0.893	0.899	0.899
L—Hippocampus	0.658	0.658	0.575	0.831	0.831
R—Hippocampus	0.59	0.59	0.57	0.809	0.809
L—Pallidum	0.959	0.959	0.125	0.017	0.017
R—Pallidum	0.624	0.624	-0.042	-0.165	-0.165
L—Putamen	0.687	0.687	0.713	0.637	0.637
R—Putamen	0.781	0.781	0.618	0.68	0.68
L—Thalamus	0.777	0.777	0.586	0.319	0.319
R—Thalamus	0.774	0.774	0.666	0.548	0.548

**Fig 3:**
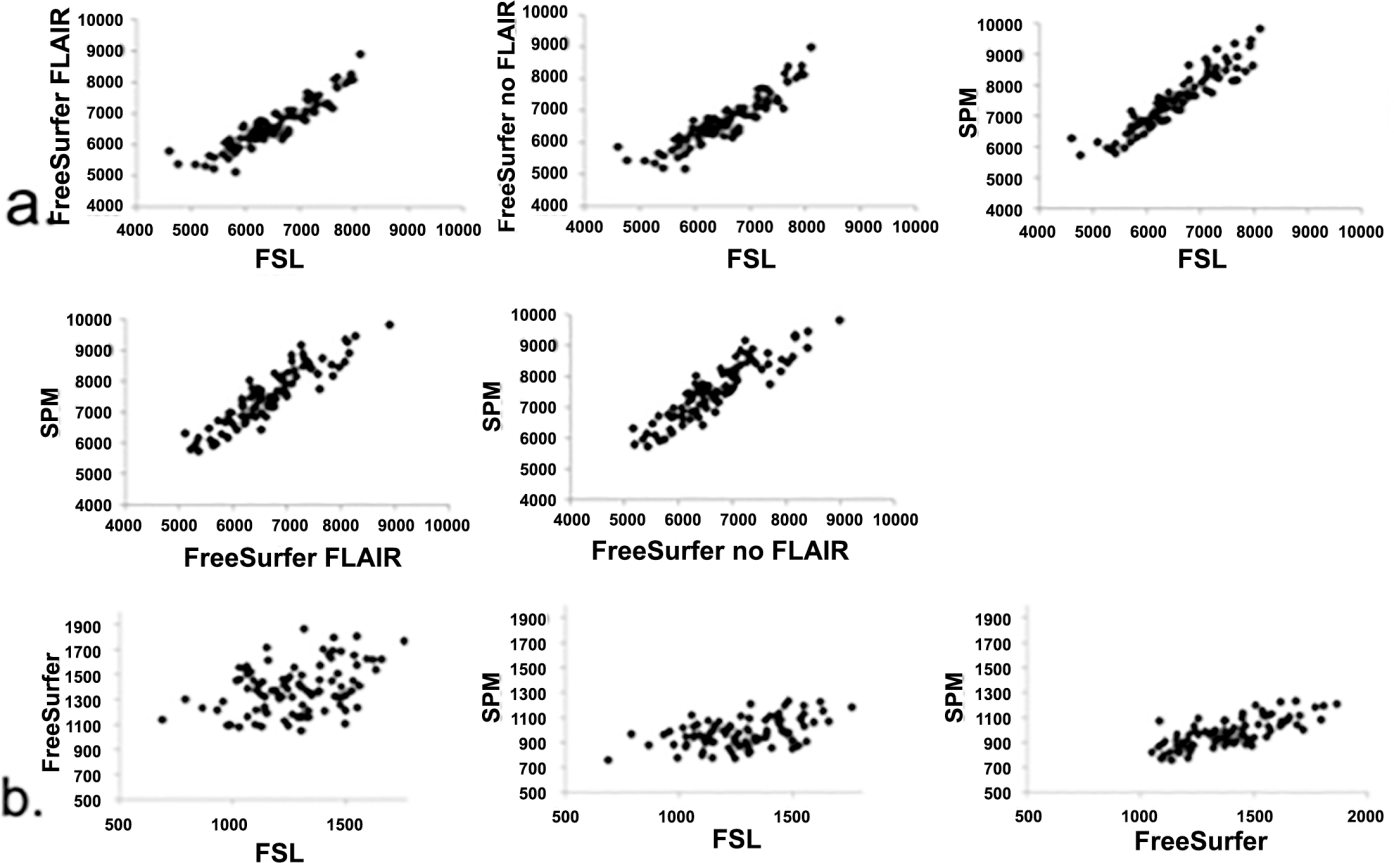
Scatterplots of the volumes of the a. L-Insula (high ICC values–see Table 4) and b. L-Amygdala (low ICC values–see Table 4).

The only DGM structure with high agreement for all methods was the caudate nucleus (bilaterally). The bilateral hippocampus showed high agreement between FreeSurfer and SPM. For thalamus volumes bilaterally, high agreement was observed between FSL and FreeSurfer, while SPM showed low ICC with both FSL and FreeSurfer. For all other DGM structures, SPM also exhibited low agreement with both FSL and FreeSurfer, with worst agreement for the left and right pallidum (all ICC < .13). There was low agreement between FSL and FreeSurfer for the bilateral amygdala (ICC < .39), hippocampus (ICC < .66), and nucleus accumbens (ICC < .66). Fig 3 provides scatterplots of regional volumes for the left insula (high ICC values) and the left amygdala (low ICC values).

Unfortunately nucleus accumbens is not available in the AAL atlas so this was not analyzed for SPM.

### Consistency of patient/control discrimination

The cortical regions were not significantly different between patients and controls with t-tests, but when using MANOVA (with age and gender included) the temporal lobes bilaterally were significantly different between MS and controls for FSL and FreeSurfer, but not for SPM.

The following DGM structures were significantly different between patients and controls with all methods when t-tests were used: bilateral thalamus, caudate nucleus, putamen, hippocampus, amygdala.

**Fig 4:**
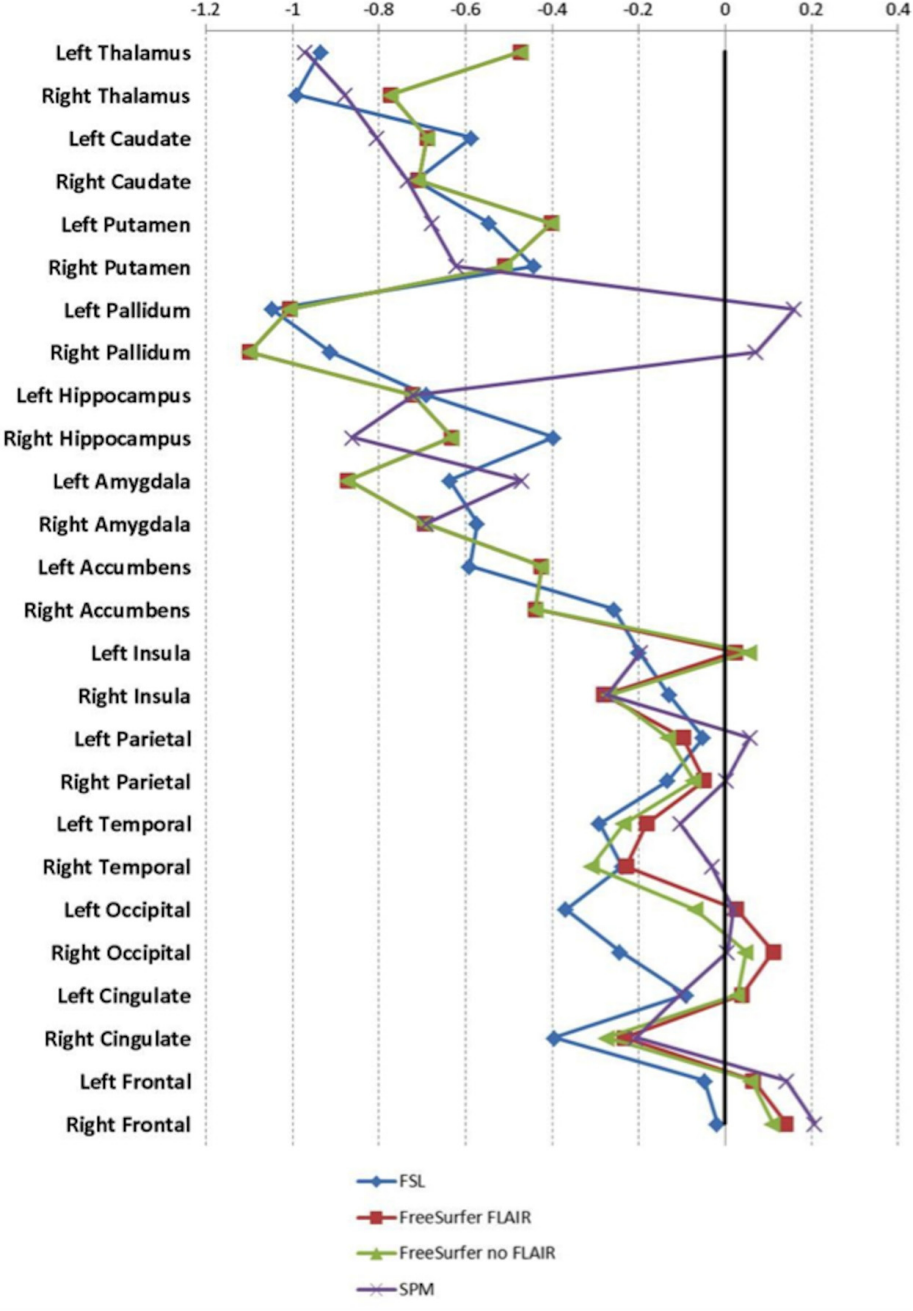
Cohen’s d.

The Cohen's d results for all methods are depicted in Fig 4, with larger effects for the DGM structures than cortical regions for all methods, except for the pallidum in SPM. The MANOVA results were similar to the t-tests results for the DGM structures.

The penalized double-loop logistic classifier revealed bilateral thalamus, pallidum and amygdala to consistently contribute to patient-control separation for all three methods.

### Consistency of association with cognition

For clarity, only correlations between volumes and cognition that were statistically significant for at least one method are discussed here. Cognition correlated moderately with EDSS scores (r = -.313, p = .002) and T1 lesion volumes (r = -.308, p = .003) and weakly with NBV (r = .218, p = .035), NGMV (r = .244, p = .018) and T2 lesion volumes (r = -.271, p = .008).

For the lobar volumes, general cognition correlated weakly (r = .206-.239) with the right insular volume with all methods, and also with the left insular volume for FSL. Significant correlations with cognition are listed in Table 5.

**Table 5:** Statistically significant correlations lobar GM volumes with cognitive domains

	Pearson’s				ANOVA (B)			
	FSL	FreeSurfer FLAIR	FreeSurfer no FLAIR	SPM	FSL	FreeSurfer FLAIR	FreeSurfer no FLAIR	SPM
L-Insula	.224*			.255*	0.239	0.199	0.177	0.27
L-Parietal						0.32	0.31	
L-Temporal				.219*	0.04*	0.031*	0.033*	0.04*
L-Occipital					0.025*			
L-Cingulate				.224*				0.095*
L-Frontal		.232*		.297*		0.028*		0.023*
R-Frontal				.269*				0.02*
R-Cingulate								
R-Occipital								
R-Temporal							0.01*	
R-Parietal								
R-Insula	.206*	.234*	.239*	.267**	0.234*	0.25*	0.247*	0.29**
L- Accumbens		.251*	251*		1.392*	1.243*	1.243*	
R- Accumbens	.244*				1.227*			
L- Amygdala					0.561*	0.68*	0.68*	
R- Amygdala	.267**				0.738**	0.826*	0.826*	1.492*
L- Caudate				.261*	0.285*			0.338**
R- Caudate				.209*	0.263J*			0.27*
L- Hippocampus	.332**				0.414**	0.302*	0.302*	0.383*
R- Hippocampus	.219*				0.249*	0.266*	0.266*	0.48*
L- Pallidum					0.709*			
R Pallidum	.229*				1.104**	0.468*	0.468*	
L- Putamen	.309**	.383**	.383**	.286*	0.294**	0.252**	0.252**	0.285**
R- Putamen	.289*	.308**	.308**	.226*	0.322*	0.264*	0.264*	0.205*
L-Thalamus	.364**			.223*	0.307**	0.137*	0.137*	0.353**
R-Thalamus	.390**			.222*	0.353**	0.166*	0.166*	0.31*

* = significant at p<0.05 level

** = significant at p < .001 level.

Among DGM, the bilateral putamen volumes correlated with cognition for all methods (r = .226 to .383). FSL yielded the largest number of significant correlations, including thalamus and hippocampus volumes bilaterally. The thalamus volumes bilaterally also significantly correlated for SPM, but not for FreeSurfer. Surprisingly, neither FreeSurfer nor SPM yielded significant correlations with hippocampus volumes.

ANOVA (with gender and age included in the model) revealed the bilateral insula and the left temporal volume as significantly correlated with cognition using all methods. Among DGM structures, the bilateral thalamus, putamen, hippocampus and the right amygdala volumes were also correlated with cognition using all methods. The left amydgala volume was also correlated with cognition when using FSL and FreeSurfer but not SPM. The other regional volumes (including right temporal lobe and bilateral pallidum) correlated with cognition for some but not all methods (Table 5).

### Voxel-wise and vertex-wise comparisons between MS and controls

**Fig 5:**
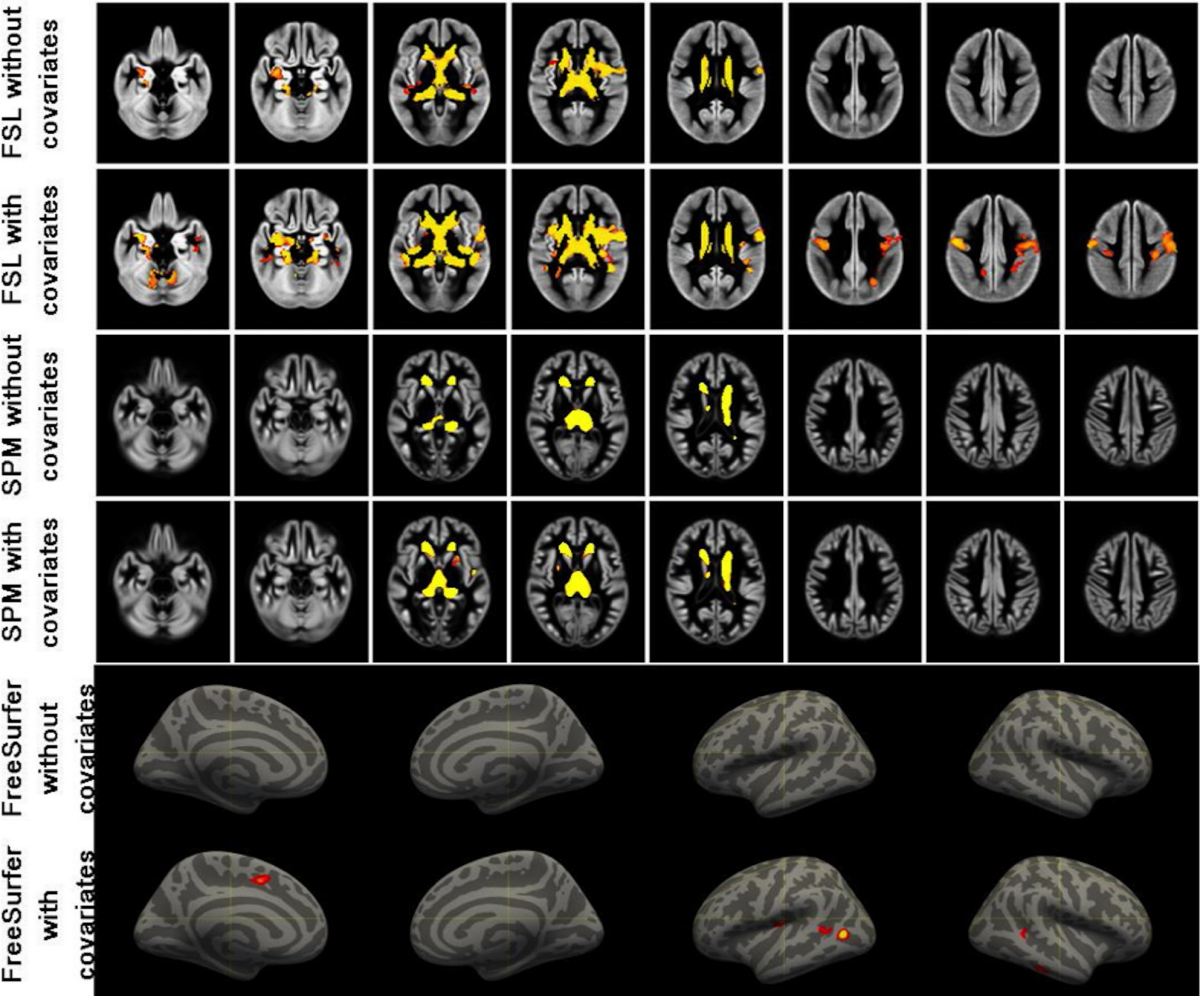
Results of FSL-VBM, SPM-VBM and vertex-wise FreeSurfer, both with and without covariates. The software package and covariate choice is indicated on the left side of the image. The covariates are age and gender. The order of the FreeSufer images is left hemisphere medial side, right hemisphere medial side, left hemisphere lateral side, right hemisphere lateral side.

FSL-VBM, with and without covariates, revealed significant clusters in the thalamus, caudate nucleus, amygdala, insula, part of the left precentral gyrus. When using covariates, also both precentral gyri and the precuneus were significantly different. SPM-VBM8 revealed thalamus and caudate nucleus bilaterally to be significantly different with and without covariates. When using covariates, also the right hippocampus, bilateral putamen and the left insula were significant. FreeSurfer vertex-wise analysis revealed no significant cortical thickness differences without covariates, but in the analysis with covariates did reveal significant clusters in the left paracentral lobule, and bilateral temporal lobes (Fig 5).

### Influence of the atlases on the agreement measures

When using the FreeSurfer parcellation on the FSL and SPM segmentation, the ICC values with FreeSurfer became very high (over 0.9) but also the ICC between FSL and SPM dramatically improved, with all ICC>0.7. However, for SPM, even when using the FreeSurfer parcellation, the agreement with FSL remained low for the right insula and the occipital lobes bilaterally. After dilating the AAL atlas the ICC impoved for the cortical structures but not for the DGM structures.

## Discussion

Our study investigated the agreement between three software packages used frequently in MS GM atrophy literature, using a large cohort of MS patients with a single disease type, of homogeneous disease duration, and matched controls. We not only analyzed the consistency of volume measurements but also the consistency of patient-controls discrimination and the consistency of correlations with cognition. Marked differences between software packages were observed, as the consistency of volume measurements was suboptimal with ICC values ranging widely. The structures with the lowest agreement were the insula, amygdala, nucleus accumbens and hippocampus, which have received a lot of interest in MS research [9,25–28].

Although the agreement was low, it may not affect patient-control discrimination at the group level, as shown by the fact that the Cohen’s d values and the combined models from the logistic classifier were comparable between methods in our study. However, the low ICC values indicate potential difficulties when addressing relations with clinical or cognitive variables, and this was confirmed by the variability across methods of correlations with overall cognition found in this study.

Previous studies report different regions as the site of GM atrophy in MS; these differences may be due to the study methodology including the choice of image analysis method used to measure the GM atrophy. We were able to confirm this in a large MS patients cohort. Differences between automated methods have already been reported in bipolar disorder [29] and ALS [30]. A previous study in MS has shown differences in whole brain atrophy measurements between software packages [31]. The reason for such strong differences between software packages is difficult to pinpoint. Another previous study evaluated the accuracy of GM segmentation in MS against a manually drawn gold-standard in a small patient group [32]. That study already indicated that the methods do not agree on the precise location of the structures. In our study due to the large sample size we were unable to produce a manual segmentation reference, so we opted for the comparison of actual automated methods used in the literature. Also in our analysis comparing lobar volumes, and not at a voxel-wise level, there is considerable variation between methods which validates the results in the aforementioned study [32]. The fact that DGM structures display larger differences between methods than cortical GM may be due, first, to the larger size of the lobes compared to the DGM structures, leading to smaller relative effects of small segmentation differences, and second, to the less conspicuous nature of the DGM borders compared to the cortical borders.

Lesion misclassification may be a major problem for techniques such as SIENAX and VBM, but not FIRST [33]. We tried to minimize the effect of the presence of white matter lesions or excessive neck slices in the image by using only images after neck-clipping and lesion-filling. Differences between the software packages could therefore stem from the segmentation itself, the atlas used, or the smoothing kernel in case of voxelwise analyses). In fact, in our study the agreement dramatically improved when using the FreeSurfer lobar parcellation on the FSL and SPM segmentation, which would indicate that the difference between software is in large part related to the atlas. This could be due to either its definition of the anatomical structures or the registration method used to apply the atlas; the latter may affect primarily smaller structures such as the pallidum, which showed poor agreement in our study. Similarly, structures such as the nucleus accumbens may have smaller differences in intensity compared to the surrounding tissue on the 3DT1 images, and will be more prone to errors in segmentation for intensity-based segmentation methods. The lack of a generally accepted gold-standard for regional atrophy measurements including all brain structures, impedes on the assessment of false negative or false positive voxels.

Still the separation between MS patients and controls was good, with some structures showing consistently large effect sizes (see Cohen’s d in Fig 4), and consistent selection of thalamus, putamen and amygdala as the most important regions for MS-control discrimination in the double-loop classifier. This points to a real disease effect, in that for early RRMS patients the DGM structures are atrophic; cortical structures may be affected at a later disease stage [25]. The thalamus has already been pointed to as different between patients and controls in MS [5,10,34–36], as well as the amygdala [37] and the putamen [8,38–40].

Although we found few differences between patients and controls in the cortex, we used the lobar cortical GM for comparison. Therefore there may be differences at the more detailed level of the gyri, as pointed out by the voxel-based analyses. Further studies with larger sample sizes in a longitudinal setting should assess the consistency at gyrus level.

For MS research the VBM analyses are run with covariates in the model, which allows for plausible results. In order to more specifically examine the influence of image processing and analysis, we also ran the analysis without covariates: for all methods more regions appeared different between patients and controls when covariates were included in the model. FSL in general detected more regions as significantly different. We were able to confirm the cortical regions already reported as different between patients and controls in a meta-analysis of VBM studies in MS [41]. Differences between results of cortical studies are apparent however, which could be due to the possible different GM atrophy evolution of different MS types, which we precluded by selecting only RRMS patients for our study. The processing errors may artificially appear to increase the differences between patients and controls by underestimating the grey matter volumes in patients with more severe pathology, and the analysis software may also be more likely to fail for these images: on half of the scans who had to be excluded from our sample the cortex had been severely underestimated with the pial surface running through the cortex; the patients were older and had higher WM lesion loads. Future studies should also look at longitudinal effects with different suitable software packages, as longitudinal measurements would provide important information for the monitoring of patients in clinical studies and may also have smaller errors [42].

Although the separation at the group level between patients and controls was relatively good for all methods, the differences between methods were not systematic, which led to differences in correlations with other measures. Given the low ICC between GM atrophy methods, correlations with clinical measures, such as cognition and EDSS scores, may be affected. We indeed observed varying correlations with overall cognition, and it is therefore clear that correlations with overall cognition reported in the literature have to be interpreted with some caution. In line with previous research, the right insula was most commonly related to cognition, whether age and gender were used as covariates or not [43] as well as the putamen bilaterally [44,45].

In conclusion, regional GM volumes obtained from these three popular image analysis methods can be very different, especially for cortical regions and to a lesser degree for DGM. While group-level separation between MS and controls is comparable between analysis methods, correlations with cognitive and clinical measures can vary, depending on the analysis method chosen. Correlations between regional GM volumes and clinical or cognitive variables in MS should be interpreted with caution.
